# Raman Lasing in a Tellurite Microsphere with Thermo-Optical on/off Switching by an Auxiliary Laser Diode

**DOI:** 10.3390/mi14091796

**Published:** 2023-09-20

**Authors:** Elena A. Anashkina, Maria P. Marisova, Vitaly V. Dorofeev, Alexey V. Andrianov

**Affiliations:** 1 A.V. Gaponov-Grekhov Institute of Applied Physics of the Russian Academy of Sciences, 46 Ulyanov Street, Nizhny Novgorod 603950, Russia; 2 Advanced School of General and Applied Physics, Lobachevsky State University of Nizhny Novgorod, 23 Gagarin Ave., Nizhny Novgorod 603022, Russia; 3 G.G. Devyatykh Institute of Chemistry of High-Purity Substances of the Russian Academy of Sciences, 49 Tropinin Street, Nizhny Novgorod 603951, Russia

**Keywords:** optical microresonator, tellurite glass, whispering gallery modes, Raman lasing, thermo-optical control

## Abstract

The generation of coherent light based on inelastic stimulated Raman scattering in photonic microresonators has been attracting great interest in recent years. Tellurite glasses are promising materials for such microdevices since they have large Raman gain and large Raman frequency shift. We experimentally obtained Raman lasing at a wavelength of 1.8 µm with a frequency shift of 27.5 THz from a 1.54 µm narrow-line pump in a 60 µm tellurite glass microsphere with a Q-factor of 2.5 × 10^7^. We demonstrated experimentally a robust, simple, and cheap way of thermo-optically controlled on/off switching of Raman lasing in a tellurite glass microsphere by an auxiliary laser diode. With a permanently operating narrow-line pump laser, on/off switching of the auxiliary 405 nm laser diode led to off/on switching of Raman generation. We also performed theoretical studies supporting the experimental results. The temperature distribution and thermal frequency shifts in eigenmodes in the microspheres heated by the thermalized power of an auxiliary diode and the partially thermalized power of a pump laser were numerically simulated. We analyzed the optical characteristics of Raman generation in microspheres of different diameters. The numerical results were in good agreement with the experimental ones.

## 1. Introduction

The generation of coherent light with the help of the well-known phenomenon of inelastic stimulated Raman scattering in photonic microresonators has been attracting great interest in recent years. Raman microresonator lasers find various applications as sensors and biosensors [1,2] and provide a promising platform for studying fundamental physical phenomena [3,4,5]. While traditional lasers with population inversion can operate at certain wavelengths belonging to the gain band, Raman lasers can potentially operate in the entire transparency window of the used material (with suitable pump lasers). Thus, Raman microlasers are highly promising for generating coherent light in spectral ranges that are poorly mastered with traditional lasers.

To expand the capabilities of Raman microlasers, special materials with large Raman gain may be used, such as tellurite glasses, which are transparent from visible wavelengths up to five microns and even longer [6]. Tellurite glasses with large third-order Kerr and Raman nonlinearities are actively used in nonlinear fiber optics for ultra-wideband light conversion [7,8,9]. Tellurite glasses also attract the attention of different research groups as materials for manufacturing microresonators of various geometries (e.g., microspheres [10], microbubbles [11], and integrated microrings [12]). To date, great progress has been made in lasing in tellurite microresonators doped with rare-earth ions [10,11,12,13,14]. However, Raman lasing in tellurite microresonators has been implemented, as far as we know, only by our team [15]. As one of the reasons, we see the insufficiently high Q-factors obtained earlier. However, this problem is technically surmountable, as we have achieved Q-factors of up to 3.7 × 10^7^ [16] (which is far from the fundamental limit for tellurite glasses). Tellurite glasses have a very wide Raman gain band, the maximum of which, depending on exact composition, can be at a frequency of 20–28 THz [17] (against 13 THz for the commonly used silica glass), which is very attractive for wideband frequency conversion. Therefore, research into and the development of tellurite glass Raman microlasers seems to be an interesting and promising direction.

When working with microresonators made of different materials (not only tellurite glasses), there arises a question of how to excite a narrow-line whispering gallery mode (WGM). When a microresonator is pumped, part of the radiation is inevitably thermalized, which leads to microresonator heating and then to a shift in resonant frequencies (due to changes in the refractive index Δ*n* = (*dn/dT*)Δ*T* and changes in the diameter via thermal expansion) [18]. Thus, it is a challenge to start nonlinear optical processes in WGM microresonators. Different ways have been proposed to overcome this challenge, such as self-injection locking [19], elect ro-optical controlling, two-wavelength pumping, thermal tuning (dynamical tuning of eigenfrequencies from lower to higher ones), scanning the frequency of a narrow-line pump laser (from higher to lower ones), and some others [20]. All of them have their own technical features, advantages, and disadvantages.

Previously, to start Raman lasing in a 30 µm tellurite microsphere, we used the method of scanning the frequency of a narrow-line pump laser, which causes mode pulling and leads to a steady state after the scanning is stopped [15]. Here, we propose an alternative way to initiate Raman generation in a tellurite microsphere pumped by a narrow-line laser at a fixed frequency. This method employs a cheap, low-power violet-blue auxiliary laser diode at 405 nm, with the help of which resonant frequencies of the WGM are thermo-optically controlled (405 nm light is strongly absorbed by tellurite glass). Thus, the usually parasitic thermo-optical nonlinearity is exploited here as a simple and cheap way to control the on/off switching of Raman lasing. It should be noted that thermo-optical effects have been previously used to control intracavity light in different regimes, including laser wavelength tuning [18,20,21,22,23,24].

We report the experimental implementation of Raman lasing at 1.8 µm in a 60 µm homemade tellurite glass microsphere pumped at 194.4 THz (~1.54 µm) with thermo-optical on/off switching with the help of a cheap 405 nm laser diode. The experimental results are supported by numerical simulations of thermo-optical effects and Raman lasing.

## 2. Materials and Methods

### 2.1. Manufacturing Tellurite Glass Microspheres

**Figure 1:**
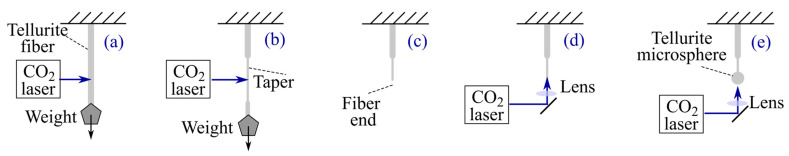
(**a**–**e**) Step-by-step illustration of the manufacturing process of tellurite microspheres.

We manufactured microspheres from tellurite filaments (coreless fibers) drawn from a melt of high-purity tellurite glass. We used an original technology for tellurite glass synthesis described, for example, in [25,26]. To form a solid microsphere at the end of the filament, we carried out the process schematically presented in Figure 1. The glass filament was softened and melted with a CO_2_ laser. This process has been described in detail in [16,26]; here, we briefly consider the main steps. First, the filament was vertically suspended with a weight at its end (Figure 1a). Second, a taper was formed by softening the filament with CO_2_ laser pulses (Figure 1b), and then it was split into two parts (Figure 1c). Next, a solid glass microsphere was formed at the tapered end under the action of CO_2_-pulsed radiation due to the surface tension force (Figure 1d,e). The size of the microspheres was controlled in the 40–100 µm range by the number of laser pulses and their energy [16].

### 2.2. Experimental Methods

Raman lasing with thermo-optical on/off switching in a tellurite microsphere was studied on a testbed previously described in [15], with key modifications concerning the integration of a violet-blue laser diode in the scheme (to be described in the section Results). To couple the continuous wave (CW) pump light and the light of an auxiliary violet-blue laser diode into a tellurite microsphere and extract the converted light from it, we used a silica fiber taper with a waist diameter of about 2 µm.

**Figure 2:**
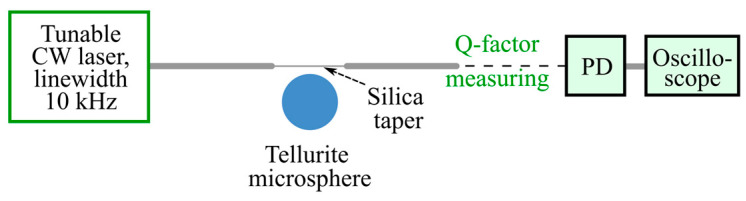
Schematic diagram of the testbed for Q-factor measuring. PD is a photodetector.

For measuring the Q-factors of the produced tellurite glass microspheres, we used a well-known dynamic method of analyzing resonant WGM dips by scanning them with a tunable narrow-line CW laser and recording them with an oscilloscope (Figure 2). The parameters were the following: scanning rate 10 GHz/s, scanning range 70 GHz, and central frequency *f*_0_ near 194 THz. To operate in a linear regime and avoid nonlinear effects leading to distortions in resonance line shapes, the pump power was decreased to 100–200 nW. The Q-factors were calculated as *Q = f*_0_/*δf*, where *δf* is the linewidth (at 1/2 level).

### 2.3. Calculation of Microsphere Parameters and Thermo-Optical Simulations

To calculate important parameters of tellurite microspheres, such as electric fields of eigenmodes and their effective volumes, we applied a previously developed and well-tested approach [26]. The eigenfrequency problem for a dielectric solid sphere is well known, and a characteristic equation for WGMs was derived from Maxwell’s equations [27]. Effective mode volumes are found using expressions for electric fields [27]. We numerically found solutions for the corresponding characteristic equation for eigenmodes; the wavelength dependence of refractive index, which was approximated from the Sellmeier formula [28], was taken into account iteratively. Moreover, we included in our model thermo-optical effects (changes in the refractive index Δ*n* = (*dn*/*dT*)Δ*T* and changes in the diameter via thermal expansion) that are of principal importance here [18]. For the tellurite glass, the coefficient of thermal expansion *α* and the thermo-optic coefficient (*dn*/*dT*) have comparable absolute values but different signs (see Table 1). Both effects should be taken into account to provide an accurate prediction of the total WGM frequency shift. To do this, we obtained steady-state temperature distributions by the finite element method (FEM) and calculated the average temperature increase in the region corresponding to the eigenmode (Δ*T_mode_*), which affects the refractive index change, and the temperature increase averaged over the whole microsphere (Δ*T_aver_*), which affects the thermal expansion. The obtained data were imported into our numerical code for finding eigenfrequencies. We found the thermal shifts by comparing “cold” and “hot” solutions.

We assumed in our calculations that we operated with the fundamental eigenmode. In this case, the effective volume is minimal, and, accordingly, the nonlinear third-order coefficient responsible for Raman lasing is maximal.

The parameters that we used for the theoretical study, including the calculation of the characteristics of the microsphere, thermo-optical simulation, simulation of thermo-optical shifts, and Raman lasing, are listed in Table 1.

### 2.4. Simulation of Raman Lasing

The theoretical investigation of Raman lasing was performed for mean electric fields using the coupled mode theory [29]:

(1) $$\frac{dA_{p}}{dt}=\left(i∆\omega_{0}-\frac{1}{2\tau_{p}}A_{p}\right)-g_{R}\frac{\omega_{p}}{\omega_{R}}{\left|A_{R}\right|}^{2}A_{p}+\sqrt{\kappa_{p}P_{p}},$$

(2) $$\frac{dA_{R}}{dt}=-\frac{1}{2\tau_{R}}A_{R}+g_{R}{\left|A_{p}\right|}^{2}A_{R},$$

where *A_p_* is the intraresonator amplitude of the electric field at the pump angular frequency *ω_p_*; *t* is the time; *A_R_* is the intraresonator electric field amplitude of the Raman wave at the angular frequency *ω_R_*; Δ*ω*_0_ is the angular frequency detuning of the pump from the nearest WGM resonance; *P_p_* is the pump power; *τ_p,R_* is the total photon lifetime at *ω_p,R_* (related to the loaded Q-factor by *Q_p,R_* = *ω_p,R_* × *τ_p,R_*); *κ_p,R_* is the coupling coefficient (we set *κ_p,R_* = 1/(2 × *τ_p,R_*)); $g_{R}=\Gamma g_{Te}c^{2}/(2n^{2}V_{R})$ is the intraresonator Raman gain coefficient; *c* is the speed of light in vacuum; $g_{Te}$ is the Raman gain for tellurite glass taken at frequency *ω_p_* − *ω_R_*; *n* is the linear refractive index; *V_R_* is the effective volume of the WGM where Raman wave is generated; and *Γ* is the overlap integral between the pump and Raman waves. The output Raman power is *P_R_* = *κ_R_*|*A_R_*|^2^.

We analyzed the steady-state Raman lasing setting *dA_p,R_*/*dt* = 0 in Equations (1) and (2). The threshold pump power *P_th_* for starting Raman lasing was found analytically from these equations in the following form

(3) $$P_{th}=\frac{1}{2\kappa_{R}\tau_{R}g_{R}}\left[\frac{1}{4\tau_{p}^{2}}+∆\omega_{0}^{2}\right].$$

## 3. Results

### 3.1. Experimental Results

**Figure 3:**
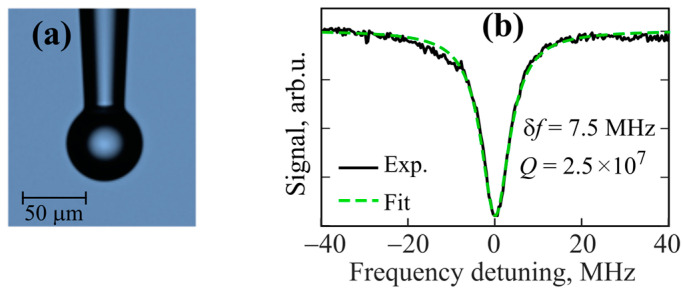
(**a**) Image of 60 µm tellurite glass microsphere obtained with optical microscope. (**b**) Resonance dip for finding Q-factor of this sample: experimentally measured (Exp) and fitted by Lorentz curve (Fit).

We manufactured a set of tellurite microspheres with diameters in the 40–100 µm range. An image of a 60 µm tellurite glass microsphere obtained with an optical microscope is demonstrated in Figure 3a. Its measured and Lorentz-fitted resonance curves are plotted in Figure 3b. The resonance dip has a width of *δf* = 7.5 MHz at a central frequency of about 194 THz corresponding to the loaded Q-factor *Q* = 2.5 × 10^7^. Note that for the produced samples, the loaded Q-factors were similar (~2–2.5 × 10^7^) and almost independent of the diameter.

The main idea of the paper is thermo-optical on/off switching of Raman lasing in a tellurite microsphere with the help of a cheap 405 nm low-power auxiliary laser diode. The mechanism of enabling and disabling Raman generation is based on a simple manipulation of detuning between the WGM frequency and the pump frequency by heating the microresonator with the blue diode. This allows for the controlling of the intracavity pump power, which can be set above or below the Raman lasing threshold, thus allowing the Raman laser to be turned on and off. Let us explain this in more detail. Let the pump laser and the auxiliary diode operate simultaneously but assume that there is no Raman generation (“hot” eigenfrequencies are far from the pump frequency). Next, the diode is switched off. This leads to the blue shift in WGMs due to the microsphere cooling. When some suitable WGM passes through the laser frequency, the intracavity pump power increases above the Raman generation threshold, and there occurs Raman lasing. Thanks to partial pump thermalization during Raman generation, the microsphere is heated, and the process can stabilize. If a slight fluctuation in the “hot” WGM frequency occurs in the blue direction, then the detuning Δω_0_ decreases. Therefore, the conditions for Raman generation will improve, Raman power will grow, and heat dissipation will increase. Thus, the microsphere will heat up, and the WGM frequency will move back. If, on the contrary, the frequency fluctuation occurs in the red direction, then the detuning Δω_0_ will increase, the pump thermalization will decrease, the microsphere will cool, and the WGM frequency will also return back. To disable Raman generation, the blue diode is switched on again. The temperature rises, and the WGM frequency is shifted further to the red-detuned side from the pump frequency. This reduces the pump efficiency, and the intracavity pump power drops below the Raman generation threshold, so the Raman generation stops. It should be noted that as the intracavity pump field decreases, its thermalized power also decreases, counteracting the effect of heating by the blue diode. However, if the power of the auxiliary diode is large enough, the red detuning is sufficient to move the WGM resonance far away from the pump frequency. Finally, to restore Raman lasing, the blue diode is switched off, and the WGM resonance returns back to the vicinity of the pump frequency, where it is self-stabilized due to pump thermalization.

**Figure 4:**
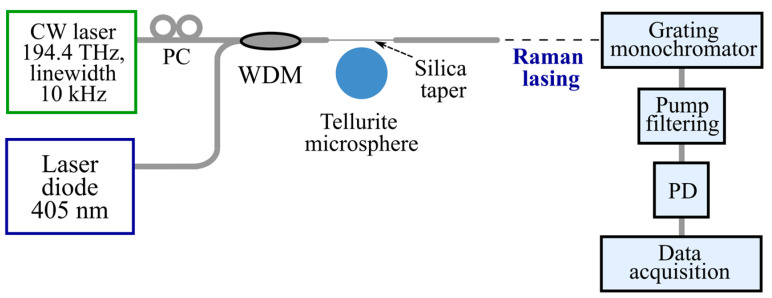
Schematic diagram of the testbed. PC is a polarization controller, WDM is a wavelength division multiplexer, and PD is a photodetector.

To demonstrate experimentally this idea of thermo-optical on/off switching of Raman lasing, a testbed was assembled (a simplified scheme is shown in Figure 4). We used a pump laser at a fixed frequency *f*_0_ = 194.4 THz to generate a Raman wave shifted by 27.5 THz. A polarization controller was used to adjust the optimal polarization of the pump wave. The pump wave was combined with the help of a wavelength division multiplexor (WDM) with the light of a violet-blue diode and was then launched into a 60 µm microsphere through a silica taper. Tellurite glass is nontransparent at a wavelength of about 400 nm (the measured transmission is almost zero [25]). Therefore, the auxiliary diode radiation (with a maximum output power of 2 mW before the fiber taper) was well absorbed in the microsphere near the equator. A Raman wave pumped at 194.4 THz could be generated if the frequency of some eigenmode was tuned to the pump frequency, and this mode was pulled. Note here that the WGM frequency should be tuned to the pump from a lower to a higher value to achieve mode pulling (similar to the conventional method of pump frequency scanning from a higher to a lower value). To record spectra of Raman lasing near 1.8 µm, we used a grating monochromator and a polycrystalline lead sulfide (PbSe) photodetector. The residual pump light was blocked by a filter to avoid photodetector damage.

**Figure 5:**
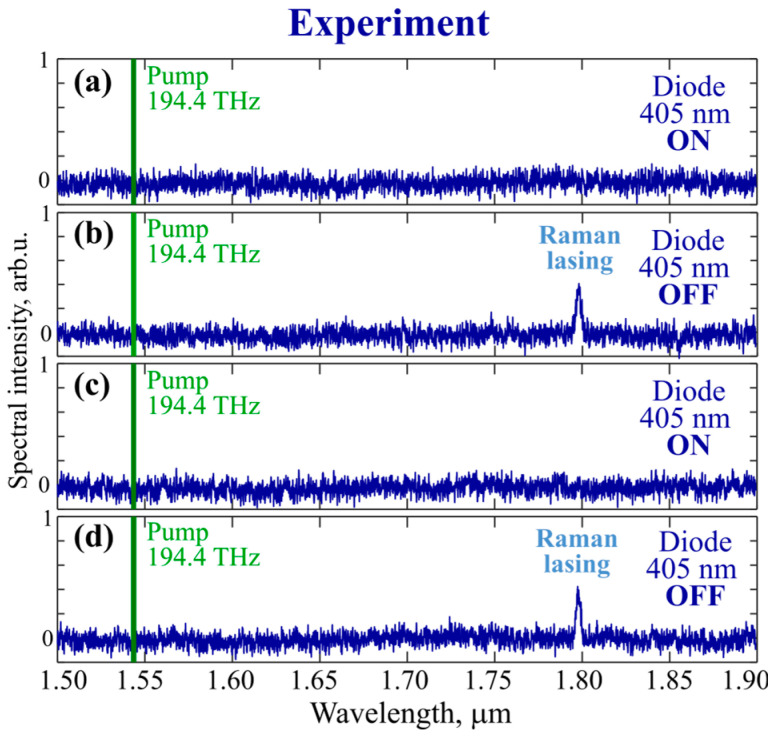
*Experimental* spectra demonstrating thermo-optical control of Raman lasing by auxiliary blue-violet laser diode. Pump laser at constant frequency of 194.4 THz operates continuously without shutdowns in (**a**–**d**). When blue-violet diode is switched on, there is no Raman lasing (**a,c**). When blue-violet diode is switched off, there is Raman lasing (**b**,**d**).

At the beginning of the experiment, we switched on the 194.4 THz pump laser at a power of 8 mW, which worked stably in the same mode during all subsequent actions described below. When the pump laser was switched on at a constant frequency, no lasing was observed since its frequency was far from the hot frequency of any high-Q WGM. Then, we switched on the violet-blue diode. The estimated thermalized power was about 1 mW at a thermal steady state (violet-blue light partially passed through the taper). Due to the thermalization of its power, the eigenmode frequencies shifted to the long-wavelength spectral region, and Raman generation, as expected, did not occur (Figure 5a) since the dynamical tuning of the eigenfrequencies was from higher to lower values.

Then, we switched off the auxiliary diode, which led to cooling of the microsphere and the frequency shift to the short-wavelength side. Thus, in this case, the shift occurred in the “correct” direction. Then, due to the mode pulling effect, the pump laser frequency turned out to be quite close to resonance, which led to Raman lasing (Figure 5b). After that, the Raman generation remained fairly stable for at least a few minutes (output power fluctuations were at the level of 1 percent). Next, we switched on the auxiliary diode again to terminate Raman generation due to the shift in the resonant pumping WGM frequency to the long wavelength side (Figure 5c). Then, we switched off the violet-blue diode again, the process of mode shifting to the blue side accompanied by pulling was repeated, and Raman generation was restored (Figure 5d). We tested several cycles of on/off switching of Raman lasing by switching off/on the auxiliary diode. The time periods between on/off switching of the diode were of the order of 10 s and longer, which was sufficient for achieving a thermal steady state (the corresponding estimates are given in Section 3.2).

Thus, we demonstrated a simple and cheap way of thermo-optically controlled on/off switching of Raman lasing in a tellurite glass microsphere, providing generation at a frequency shifted by a large value of 27.5 THz from the pump frequency.

Note that the thermo-optically controlled Raman generation was also realized in other microsphere samples with diameters of 40–80 µm. For large 90–100 µm microspheres, Raman lasing was not observed. An explanation of this fact will be provided in Section 3.3.

### 3.2. Numerical Simulation of Thermo-Optical Processes

**Figure 6:**
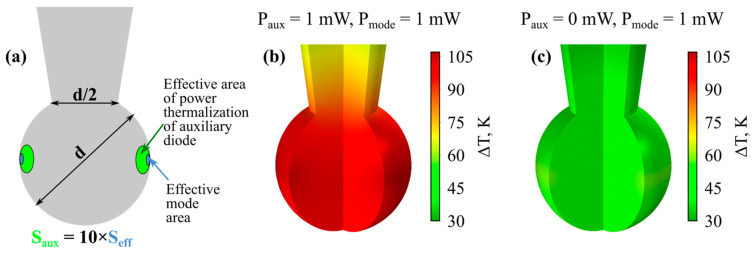
*Numerical simulation*. (**a**) Microresonator geometry used in FEM simulation of thermo-optical problem. *S_eff_* is effective mode area where partial thermalization of 194.4 THz pump occurs; *S_aux_* is effective area where thermalization of violet-blue laser diode occurs. Temperature increase for simultaneous thermalization of pump power and violet-blue diode power (**b**) and for thermalization only of pump power (**c**) calculated for 60 µm microsphere.

To theoretically support the experimentally observed thermo-optical processes, we performed numerical FEM simulations using a realistic model. The used geometry is shown in Figure 6a. A boundary condition of natural convection in air was used on all surfaces. We assumed that partial thermalization of the power of a single-mode pump laser occurs in the volume corresponding to the effective volume of an unperturbed fundamental eigenmode with effective area *S_eff_* (*S_eff_ ≈ V_eff_*/(πd)), and thermalization of the power of an auxiliary violet-blue multimode diode occurs in a region with a larger area *S_aux_*. We verified that *S_aux_* can be varied over a wide range without significantly affecting temperature fields (for the same thermalized power). In the simulations presented below, we set *S_aux_* = *S_eff_* × 10. An example of the temperature distribution when the thermalized powers of the pump laser and the auxiliary diode are equal to 1 mW in a 60 µm microsphere is shown in Figure 6b. This case qualitatively corresponds to the experimental situation when both the pump laser and the violet-blue diode are switched on (Figure 5a,c). The temperature distribution when the violet-blue diode is switched off and only the pump laser operates is shown in Figure 6c. This case qualitatively corresponds to the experimental situation in Figure 5b,d. Numerical FEM simulations of nonstationary thermal problems show that, for a 60 μm microsphere, the characteristic thermal relaxation time for the switching process is about 20 ms.

**Figure 7:**
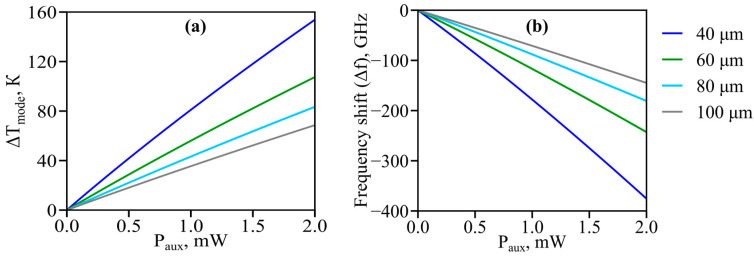
*Numerical simulation*. Temperature increase averaged over the effective area of fundamental WGMs (**a**) and WGM frequency shifts (**b**) versus power of auxiliary violet-blue laser diode for microspheres with diameters 40, 60, 80, and 100 µm.

Then, we calculated the temperature increase depending on the thermalized power of the auxiliary diode *P_aux_* in microspheres with different diameters *d*. The corresponding results averaged over the effective mode (Δ*T_mode_*) are plotted in Figure 7a. The temperature increase averaged over the whole solid microsphere is ~10% lower than Δ*T_mode_*.

Next, we simulated the frequency shift in eigenmodes as a function of the thermalized power of auxiliary diode *P_aux_* for microspheres of different sizes. The results are presented in Figure 7b. It is seen that even low diode powers of the order of a few hundred µW provide a sufficiently high thermal tuning range for eigenfrequencies, which is sufficient for thermo-optical on/off switching of nonlinear effects due to mode pulling as was demonstrated in our experiment. The smaller the microsphere, the stronger the thermal effects. Moreover, we verified that *S_aux_* can be varied in a wide range without significant impact on thermal WGM shifts. For example, the maximum thermal shift changes only by 0.7% for *S_aux_* = *S_eff_* × 50 (compared with the cases considered above for *S_aux_* = *S_eff_* × 10).

Note that Figure 7b reflects the results of calculations within the full model where “cold” and “hot” frequencies are found numerically from the characteristic equation [27]. To understand the roles of thermal expansion and refractive index changes, we present simple analytical estimates. Let us write an approximate resonance condition for WGMs: *mλ_m_* ≈ *πdn*, where *λ_m_* = *c*/*f_m_* is the wavelength of a WGM with index *m*. By varying both sides of this equation, we obtain the expression for the thermally induced WGM frequency shift: Δ*f_m_* ≈ −*f_m_*(Δ*d*/*d* + Δ*n*/*n*) = −*f_m_*(αΔ*T_aver_* + (*dn*/*dT*)(1/*n*)Δ*T_mode_*). Using the data from Table 1 and the obtained results according to which the difference between Δ*T_mode_* and Δ*T_aver_* does not exceed 10%, we find that the contribution of thermal expansion is more than three times larger than the contribution related to the refractive index changes for the examined tellurite glass.

Thus, the thermo-optical simulation confirmed that the application of a low-power auxiliary laser diode, whose radiation is well absorbed by the microsphere material, is indeed a robust, cheap, and simple way to excite the WGM due to the mode pulling effect in a nonlinear regime.

### 3.3. Theoretical Study of Raman Lasing

**Figure 8:**
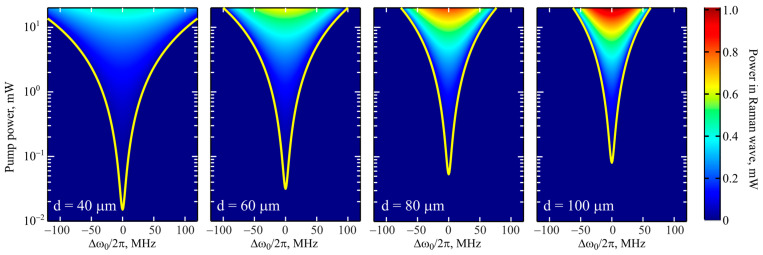
*Numerical simulation*. Output power in Raman wave as a function of two variables: detuning and pump power for different microsphere diameters. Yellow lines indicate thresholds for Raman lasing.

We also theoretically studied Raman lasing in the framework of the model described by the system of Equations (1) and (2). The parameters used in the calculations are given in Table 1. Since, in our experiments, the Q-factors practically did not depend on the size of the microspheres, we also took a constant value in the calculations. The calculated powers in the Raman wave depending on the detuning and the pump power for various microsphere diameters are demonstrated in Figure 8. The yellow lines indicate the Raman lasing thresholds obtained with the expression (3). From this expression, it can be readily found that for the same detuning, the threshold pump powers are proportional to the effective mode volume:

(4) $$P_{th}\sim V_{R}\left[\frac{1}{4\tau_{p}^{2}}+∆\omega_{0}^{2}\right].$$

The smaller the microsphere, the lower the threshold. This explains the fact that we experimentally observed Raman lasing in microspheres with a diameter smaller than 80 µm and did not observe it in the 90–100 µm samples. For relatively large microspheres, Raman lasing is achieved only at small detunings (which is not always easily achieved experimentally).

**Figure 9:**
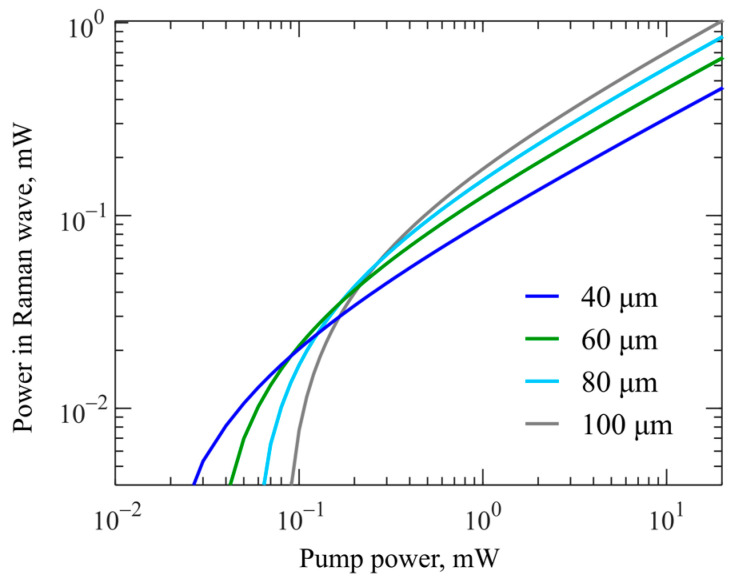
*Numerical simulation*. Output power in Raman wave as a function of pump power at zero detuning for different microsphere diameters.

We also note the interesting and not obvious fact that the maximum power in the Raman wave is achieved for large microspheres at a pump power of the order of 1 mW and higher, but the detuning should be sufficiently small (Figure 8). For more clarity, we plotted the dependences of the power in the Raman wave on the pump power at zero detuning, i.e., at exact resonance (Figure 9). It is seen that, although the thresholds for small microspheres are reached first, the Raman wave power grows faster for large microspheres at low pump powers. At a pump power of about 100–200 µW, the output powers are quite close; starting from these values, the larger the microsphere diameter, the greater the power in the Raman wave.

## 4. Discussion and Conclusions

We demonstrated experimentally a robust, simple, and cheap way of thermo-optically controlled on/off switching of Raman lasing in a tellurite glass microsphere for the first time and, to the best of our knowledge, for tellurite microresonators. With a constantly operating narrow-line pump laser, on/off switching of the auxiliary 405 nm laser diode led to off/on switching of Raman generation. The choice of tellurite glass as a Raman medium is justified by the large Raman gain and large Raman frequency shift in such glasses. We experimentally achieved Raman lasing at a wavelength of 1.8 µm with a frequency shift of 27.5 THz from the 1.54 µm narrow-line pump in a 60 µm tellurite glass microsphere with a Q-factor of 2.5 × 10^7^. This Raman shift is twice the Raman shift in a silica microsphere [29] and almost thrice as large as the Raman shift in a chalcogenide As_2_S_3_ microsphere [30,31]. The proposed simple way to start Raman lasing in a tellurite microresonator with a low-power violet-blue auxiliary laser diode without the need to tune the wavelength of the pump laser or other complicated schemes allows one to use a cheap narrow-band pump laser (for example, conventional distributed feedback (DFB) laser diodes), which makes the whole scheme very simple and cheap. The proposed approach has an obvious limitation in the modulation speed of the Raman laser (tens of milliseconds on/off switching time); however, such modulation may still be useful, for example, in systems with lock-in detection instead of mechanical choppers.

We also performed theoretical studies to support the experimental results. We carried out a FEM simulation of temperature distribution in microspheres heated by the thermalized power of an auxiliary diode and the partially thermalized power of a pump laser. We found thermal shifts in eigenmodes caused by changes in the refractive index Δ*n* = (*dn/dT*)Δ*T* and changes in diameter via thermal expansion. The smaller the microsphere, the stronger the thermal effects. The thermal shift is sufficient for switching on/off the nonlinear light conversion due to mode pulling. We analyzed the optical characteristics of Raman generation in microspheres of different diameters. The smaller the microsphere, the lower the pump power threshold. This explains why we experimentally observed Raman lasing in microspheres with a diameter smaller than 80 µm and did not observe it in the 90–100 µm samples.

## Figures and Tables

**Table 1:** Simulation parameters.

Parameter	Symbol	Value
Q-factor	*Q*	2.5 × 10^7^
Microsphere diameter	*d*	40, 60, 80, 100 µm
Effective mode volume for different *d:*	*V_R_*	
*V_R_* for *d* = 40 µm		715 µm^3^
*V_R_* for *d* = 60 µm		1506 µm^3^
*V_R_* for *d* = 80 µm		2559 µm^3^
*V_R_* for *d* = 100 µm		3861 µm^3^
Overlap integral between the pump and Raman waves	*Γ*	0.7
Pump angular frequency	*ω_p_*	2π × 194.4 THz
Raman wave angular frequency	*ω_R_*	2π × 166.9 THz
Raman gain for bulk tellurite glass	*g_Te_*	3.82 × 10 ^–12^ m/W
Thermal expansion coefficient	*α*	14.2 × 10^–6^ K^–1^
Thermo-optical coefficient	*dn/dT*	–8 × 10^–6^ K^–1^
Glass thermal conductivity	*k*	1.2 W/(m·K)
Glass density	*ρ*	5940 kg/m^3^
Glass heat capacity at constant pressure	*c_p_*	370 J/(kg·K)

## Data Availability

Data underlying the results presented in this article may be obtained from the authors upon reasonable request.
